# Fibrinogen-to-prealbumin ratio: A new prognostic marker of resectable pancreatic cancer

**DOI:** 10.3389/fonc.2023.1149942

**Published:** 2023-03-27

**Authors:** Chengqing Li, Zhiyao Fan, Wenyi Guo, Feng Liang, Xincheng Mao, Jiahao Wu, Haodong Wang, Jianwei Xu, Dong Wu, Han Liu, Lei Wang, Feng Li

**Affiliations:** ^1^ Department of Pancreatic Surgery, General Surgery, Qilu Hospital, Cheeloo College of Medicine, Shandong University, Jinan, China; ^2^ Department of General Surgery, Feicheng People’s Hospital, Taian, China; ^3^ Department of Hepatobiliary Surgery, General Surgery, Qilu Hospital, Cheeloo College of Medicine, Shandong University, Jinan, China

**Keywords:** fibrinogen-to-prealbumin ratio, tumor marker, resectable pancreatic cancer, nomogram, prognosis

## Abstract

**Background:**

The fibrinogen-to-prealbumin ratio (FPR), a novel immune-nutritional biomarker, has been reported to be associated with prognosis in several types of cancer, but the role of FPR in the prognosis of resectable pancreatic cancer has not been elucidated.

**Methods:**

A total of 263 patients with resectable pancreatic cancer were enrolled in this study and were randomly divided into a training cohort (n = 146) and a validation cohort (n = 117). Receiver operating characteristic curve (ROC) was used to calculate the cut-off values of immune-nutritional markers. The least absolute shrinkage and selection operator (LASSO) regression and multivariate Cox regression were performed in the training cohort to identify the independent risk factors, based on which the nomogram was established. The performance of the nomogram was evaluated and validation by the training and validation cohort, respectively.

**Results:**

The optimal cutoff value for FPR was 0.29. Multivariate analysis revealed that FPR, controlling nutritional status (CONUT), carbohydrate antigen 19-9 (CA19-9), carcinoembryonic antigen (CEA), and tumor node metastasis (TNM) stage were independent predictors of overall survival (OS). The nomogram was established by involving the five factors above. The C-index of the training cohort and validation cohort were 0.703 (95% CI: 0.0.646-0.761) and 0.728 (95% CI: 0.671-0.784). Decision curve analysis and time-dependent AUC showed that the nomogram had better predictive and discriminative ability than the conventional TNM stage.

**Conclusion:**

FPR is a feasible biomarker for predicting prognosis in patients with resectable pancreatic cancer. The nomogram based on FPR is a useful tool for clinicians in making individualized treatment strategies and survival predictions.

## 1 Introduction

Pancreatic cancer is one of the most lethal malignant tumors of the digestive system, and its 5-year relative survival for pancreatic cancer is only 11% (1). According to statistics from the National Cancer Institute, the incidence of pancreatic cancer has increased in recent years and has become the third leading cause of cancer death in the United States (1). Unlike other malignancies, conventional systemic chemotherapy and radiotherapy had poor clinical efficacy in patients with pancreatic cancer (2–5). Radical resection remains the only potentially curative treatment (6). However, the prognosis after radical resection not completely parallel to tumor node metastasis (TNM) stage and serological tumor markers (7). These indicators only were focused on tumor parameters and disregard the comprehensive evaluation of patients. Therefore, more new markers are needed to predict the prognosis of patients with pancreatic cancer after radical resection.

Immune-nutritional markers are drawing increasing attention for their prognostic value in recent years. Accumulating evidence shows that inflammation and malnutrition are associated with a poor prognosis in cancer patients (8, 9). Many immune-nutritional markers, such as neutrophil-to-lymphocyte ratio (NLR) (8, 9), fibrinogen-to-albumin ratio (FAR) (10), prognostic nutritional index (PNI) (11) and controlling nutritional status (CONUT) (12) have been reported to associated with prognosis for pancreatic cancer.

Recent studies have reported that fibrinogen-to-prealbumin ratio (FPR) can serve as a new prognostic marker in a variety of cancers, including hepatocellular carcinoma, colorectal cancer and esophageal cancer (13–15). Prealbumin is an important serum nutritional and inflammatory marker (16, 17). Low presurgical levels of prealbumin have been shown to be strongly correlated with a poor prognosis (18, 19). Compared with albumin, it has a shorter half-life and is less susceptible to interference from parenteral nutrition (20). Therefore, it is more sensitive to changes in the body’s nutritional status and is regarded as a marker for prognostic monitoring of patients (21, 22). Fibrinogen is a soluble plasma glycoprotein that synthesized by the liver and play a key role in blood coagulation and thrombogenesis (23). Through the participation of thrombin, fibrinogen can be converted into fibrin (24). Fibrinogen exists widely in the extracellular matrix of tumors, participates in the composition of the tumor microenvironment, and is closely related to the biological behavior of tumors (25). Some studies had reported that fibrinogen can be used as an independent factor that affects the prognosis of malignant tumors, elevated plasma fibrinogen is considered to be associated with a poorer prognosis (26–28).

However, few studies have illustrated the correlation between FPR and resectable pancreatic cancer. Therefore, this retrospective study was designed to explore the prognostic value of FPR in patients with pancreatic cancer undergoing radical resection and further develop a novel nomogram model. The established nomogram can provide more precise evaluation for the survival of resectable pancreatic cancer patients and assist the clinicians in therapeutic decision making and individual management of patients.

## 2 Methods

### 2.1 Patients and study design

**Figure 1:**
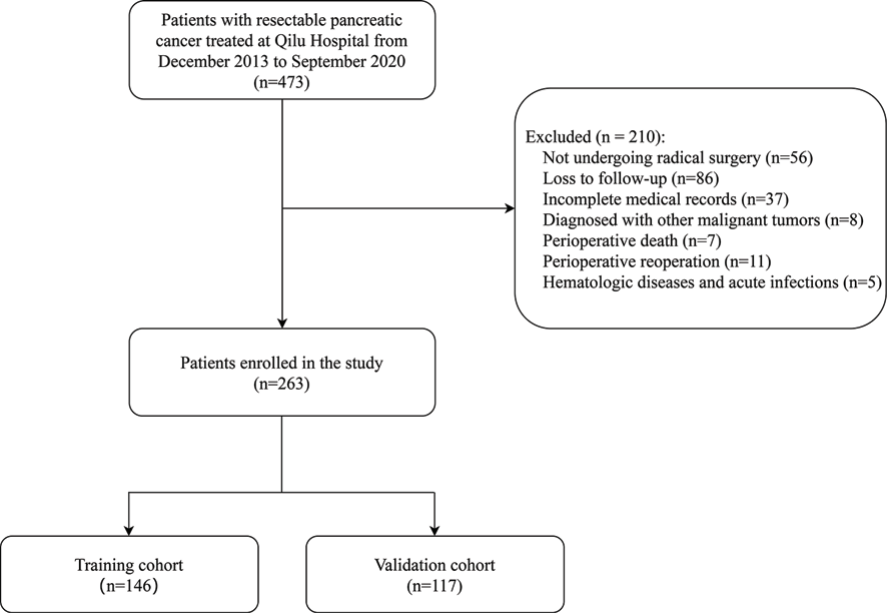
The flowchart of enrolled patients.

This study retrospectively analyzed 473 patients with resectable pancreatic cancer who were treated at Shandong University Qilu Hospital from December 2013 to September 2020. Preoperative resectability of tumors was assessed according to the National Comprehensive Cancer Network (NCCN) Clinical Practice Guideline (29). All enrolled patients were diagnosed based on histological or cytological evidence, and no evidence of distant metastasis was found through preoperative examination. None of the patients received neoadjuvant therapy before surgery. The choice of surgical approach depends on the location of the tumor, with pancreaticoduodenectomy for pancreatic head cancer and distal pancreatectomy for pancreatic body and tail cancer. The exclusion criteria were as follows: (1) patients did not undergo radical resections;(2) Patients who were lost to follow-up; (3) patients with incomplete medical records; (4) patients with other primary malignancies; (5) Patients who died in the perioperative period (within 30 days) or received perioperative reoperation; (6) patients had hematologic diseases and acute infections. After screening, this retrospective study enrolled 263 patients (**Figure 1**).

This study was carried out according to the principles of the Declaration of Helsinki. All included patients were over 18 years old. The study was approved by the Ethics Committee of Qilu Hospital of Shandong University and patient have signed informed consent upon admission.

### 2.2 Data collection

The analyzed data were collected from the medical records of Qilu Hospital of Shandong University. Data on sex, age, body mass index (BMI), hypertension, diabetes, jaundice and postoperative hospitalization stays were collected for each patient. The resected tissues were sent for histopathological examination to determine histopathology characteristics, including tumor differentiation, regional lymph node metastasis, perineural invasion, and vascular cancer embolus. The TNM stage of each patient was evaluated according to the 8th Edition American Joint Committee on Cancer (AJCC) (30). Tumors with poorly differentiated components were considered “poor”. Preoperative tumor markers and serological indicators were obtained by last venous blood within 7 days before surgery. Tumor markers that were extracted for data analysis were carbohydrate antigen 19-9 (CA19-9), carcinoembryonic antigen (CEA) and CA125. Preoperative serological indicators including hemoglobin, white blood cell counts, neutrophil counts, lymphocyte counts, platelet counts, total cholesterol, albumin, prealbumin, fibrinogen were collected. Five combinations of immune-nutritional markers examined were the FPR, FAR, NLR, PNI, and CONUT. These immune-nutritional markers were calculated according to previous reports (10–12, 31, 32). Detailed calculations of were given as following: FPR= fibrinogen/prealbumin; FAR= fibrinogen/albumin; NLR= neutrophil/lymphocyte; PNI=10*serum albumin (g/dL) + 0.005*lymphocyte count (cells/mm^3^). CONUT score was calculated based on serum albumin, lymphocyte count, and total cholesterol, and a detailed calculation process is shown in **Supplementary Table 1**. Postoperative pancreatic fistula (POPF) was defined based on the 2016 International Study Group of Pancreatic Fistula (ISGPF) definition and classification, grade B and C POPF are classified as clinically relevant POPF (CR-POPF) (33).

### 2.3 Follow-ups

The primary endpoint of this study was overall survival (OS), which was defined as the time between the date of diagnosis and the date of death due to any reason. Survival data were obtained mainly by telephone follow-up. Follow-up began at the time of diagnosis, and the terminal date was December 2021 or death.

### 2.4 Statistical methods

For measurement data, the Kolmogorov-Smirnov and Shapiro-Wilk normality tests were used to evaluate its normality. Normally distributed continuous variables were described by the mean ± standard deviation (SD) and were analyzed by Student’s t-test. Non-normally distributed continuous variables were described by the median and the interquartile range (IQR), and were analyzed by Wilcoxon rank sum test. Categorical variables are were described as percentages (%), and were analyzed by Pearson Chi square or Fisher’s exact tests. A Kaplan-Meier survival curve with a 95% confidence interval (CI) was used for analysis of OS. Univariate and multivariate analysis were separately performed based on the Cox proportional hazards regression model to calculate the hazard ratio (HR) of death. Least absolute shrinkage and selection operator (LASSO) regression and Cox regression model were performed to analyze risk factors that affect prognosis. A nomograph was constructed based on the results of variable screening. The concordance index (C-index) and calibration curves were used for validation of the nomogram. Decision curve analysis (DCA) and time-dependent area under curve (AUC) were performed to assess clinical benefit of the nomogram. X-tile 3.6.1 (Robert L Camp, M.D., Ph.D., Yale University, USA) was used to generate the optimal cut-off value to stratify patients at different risk. Descriptive analysis, univariate and multivariate Cox regression were performed using SPSS 26 (SPSS, Inc., Chicago, IL). LASSO regression, nomogram, C-index, calibration curves, DCA, time-dependent AUC and survival curve were performed using R studio version 3.6.1(R studio, Boston, Massachusetts). All P values were two-sided, and P<0.05 was considered statistically significant.

## 3 Results

### 3.1 Baseline characteristics of enrolled patients

A total of 263 patients were included in this retrospective study and the median survival time of them was 20.7 months. The baseline characteristics of included patients are summarized in **Table 1**. In the overall, the mean age of patients was 59.6 ± 9.7 years, and 96 (36.5%) patients were females. 162 (61.6%) patients had tumors located in the head of the pancreas, and 101 (38.4%) patients had tumors located in the body or tail of the pancreas. Preoperative imaging showed that 77(29.3%) patients with tumor diameter larger than 4 cm. According to AJCC 8^th^ TNM stage, 131 (49.8%) patients were stage I, 124 (47.1%) patients were stage II, and 8 (3.0%) patients were stage III. In addition, 88 (33.5%) patients had jaundice and 31 (11.8%) patients underwent preoperative percutaneous transhepaticcholangial drainage (PTCD). Enrolled patients were randomly assigned to the training group (146 patients) and the validation group (117 patients) at the ratio 5:4. There were no significant differences between the two groups, suggesting that the data from these two cohorts had a relatively strong homogeneity.

**Table 1:** Baseline characteristics of enrolled patients.

Characteristics	Total (n=269)	Training cohort (n=149)	Validation cohort (n=120)	P value
Age (years), Mean± SD	59.6 ± 9.7	59.5 ± 8.9	59.7 ± 10.6	0.888
Gender (Male/Female)	167/96	95/51	72/45	0.555
BMI (≥24 kg/m^2^/<24 kg/m^2^)	85/178	51/95	34/83	0.312
Location (Head/Body and tail)	162/101	88/58	74/43	0.622
Tumor size (≥4cm/<4cm)	77/186	44/102	33/84	0.732
Jaundice (Yes/No)	88/175	44/102	44/73	0.202
Hypertension (Yes/No)	49/214	23/123	26/91	0.181
Diabetes (Yes/No)	62/201	32/114	30/87	0.480
AJCC 8th TNM stage (I/II/III)	131/124/8	73/69/4	58/55/4	0.950
Hemoglobin (g/L), Median (IQR)	133(122-142)	133(123-141)	133(119-144)	0.658
White blood cell (10^9^/L), Median (IQR)	5.49(4.57-6.89)	5.59(4.63-7.30)	5.46(4.50-6.52)	0.278
Blood platelet (10^9^/L), Median (IQR)	215(181-271)	210(179-258)	222(182-284)	0.341
Neutrophil (10^9^/L), Median (IQR)	3.41(2.64-4.51)	3.38(2.52-4.69)	3.42(2.68-4.31)	0.740
Lymphocyte (10^9^/L), Median (IQR)	1.40(1.08-1.75)	1.43(1.05-1.82)	1.34(1.10-1.71)	0.606
Total cholesterol (mmoI/L), Median (IQR)	4.71(3.87-5.22)	4.63(3.92-5.26)	4.76(3.81-5.19)	0.962
Pre-albumin (mg/dL), Mean± SD	19.8 ± 5.9	20.0 ± 5.8	19.5 ± 6.0	0.428
Albumin (g/L), Median (IQR)	42.8(39.9-44.8)	42.9(40.8-45.0)	42.3(39.1-44.8)	0.446
Fibrinogen (g/L), Median (IQR)	3.43(3.02-3.98)	3.41(3.04-3.96)	3.46(2.94-4.15)	0.781
CA199 (≥37U/ml/<37U/ml)	190/73	101/45	89/28	0.215
CEA (≥5ng/ml/<5ng/ml)	84/179	46/100	38/79	0.867
CA125(≥35U/ml/<35U/ml)	49/214	30/116	19/98	0.372
Lymph node metastasis (Yes/No)	77/186	40/106	37/80	0.454
Laparoscopic surgery (Yes/No)	65/198	38/108	27/90	0.581
PTCD (Yes/No)	31/232	18/128	13/104	0.761

IQR, interquartile range; BMI, body mass index; AJCC, American Joint Committee on Cancer; TNM, tumor node metastasis; SD, standard deviation; CA, carbohydrate antigen; CEA, carcinoembryonic antigen; PTCD, percutaneous transhepaticcholangial drainage.

### 3.2 Cut-off values of FPR and other immune-nutritional markers

Cut-off values of FPR and other immune-nutritional markers were calculated through receiver operating characteristic (ROC) curves with Youden’s Index correction by the “SurvivalROC” package of R studio software. This package repeatedly tested all cut-off values to find the optimal which achieved the maximum log-rank statistic. The survival curve of each immune-nutritional marker was obtained by the Kaplan-Meier survival analysis (**Supplementary Figure 1**). ROC curve analysis respectively showed that the optimal cutoff value for FPR was 0.29, for FAR was 0.12, for NLR was 5.2, for PNI was 45.7 and for CONUT was 4. The timeROC plot showed that FPR had comparable prognostic accuracy with other immune-nutritional markers (**Supplementary Figure 2**). Based on the cut-off value determined by ROC curve and related standards, there were 40 (15.2%) patients with positive FPR (≥0.29), 28 (10.6%) patients with positive FAR (≥0.12), 25 (9.5%) patients with positive NLR (≥5.2), 61 (23.2%) patients with positive PNI (≤46), 19 (7.2%) patients with positive CONUT(≥4).

### 3.3 Correlations between FPR and clinicopathological characteristics

**Figure 2:**
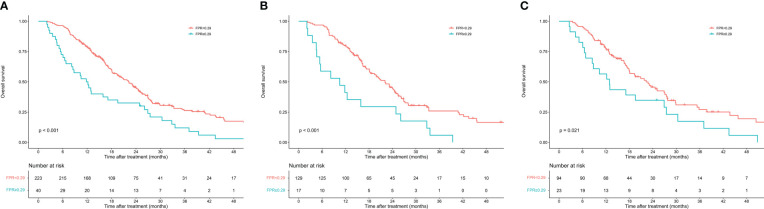
Kaplan–Meier curves of overall survival in patients with resectable pancreatic cancer stratified by fibrinogen-to-prealbumin ratio. **(A)** Total cohort **(B)** Training cohort **(C)** Validation cohort.

The enrolled patients were stratified into two groups by the optimal cutoff value of FPR: a low FPR group (FPR<0.29, n=223) and a high FPR group (FPR≥0.29, n=40). Differences in clinicopathological characteristics between subgroups were shown in **Table 2**. In total, the high FPR group had a significantly higher proportion of jaundice (55% vs 29.6%, P=0.002), pancreatic head tumors (80% vs 58.3%, P=0.009), and CA199≥37U/ml (87.5% vs 69.5%, P=0.019) than the low FPR group. Furthermore, patients with high FPR tend to have longer hospital stays (15 days vs 13 days, P=0.036) and a higher rate of CR-POPF (35% vs 15.2%, P=0.003). According to the Kaplan-Meier survival curves, in total cohort, the median survival time of patients with low FPR was significantly longer than that of patients with high FPR (22.3 months vs 11.8 months, P<0.001). High FPR was also found to be associated with poor prognosis in both the training and validation cohorts (**Figure 2**).

**Table 2:** Correlations between FPR and clinicopathological characteristics.

Characteristics	Total cohort			Training cohort			Validation cohort		
	FPR≥0.29 (n=40)	FPR<0.29 (n=223)	P Value	FPR≥0.29 (n=17)	FPR<0.29 (n=129)	P Value	FPR≥0.29 (n=23)	FPR<0.29 (n=94)	P Value
Age (years), Mean± SD	59.8 ± 9.5	59.6 ± 9.8	0.910	58.6 ± 6.9	59.6 ± 9.2	0.651	60.6 ± 11.1	59.5 ± 10.6	0.644
Gender (Male/Female)	26/14	141/82	0.830	12/5	83/46	0.612	14/9	58/36	0.941
BMI (≥24 kg/m^2^/<24 kg/m^2^)	9/31	73/150	0.149	4/13	47/82	0.294	5/18	29/65	0.388
Jaundice (Yes/No)	22/18	66/157	0.002	8/9	36/93	0.106	14/9	30/64	0.010
PTCD (Yes/No)	5/35	26/197	0.879	2/15	16/113	0.940	3/20	10/84	0.742
Hypertension (Yes/No)	6/34	43/180	0.522	3/14	20/109	0.820	3/20	23/71	0.237
Diabetes (Yes/No)	11/29	51/172	0.525	4/13	28/101	0.864	7/16	23/71	0.557
Location (Head/Body and tail)	32/8	130/93	0.009	12/5	76/53	0.355	20/3	54/40	0.009
Tumor size (≥4cm/<4cm)	9/31	68/155	0.306	3/14	41/88	0.232	6/17	27/67	0.801
Tumor differentiation (Poor/Well)	16/24	62/161	0.120	7/10	36/93	0.259	9/14	26/68	0.282
AJCC 8th TNM stage (I/II/III)	20/17/3	111/107/5	0.192	9/7/1	64/62/3	0.451	11/10/2	47/45/2	0.307
Perineural invasion (Yes/No)	9/31	82/141	0.081	3/14	50/79	0.089	6/17	32/62	0.465
Vascular cancer embolus (Yes/No)	5/35	47/176	0.210	1/16	29/100	0.111	4/19	18/76	0.847
Lymph node metastasis (Yes/No)	14/26	63/160	0.388	6/11	34/95	0.437	8/15	29/65	0.716
CA199 (≥37U/ml/<37U/ml)	35/5	155/68	0.019	14/3	87/42	0.211	21/2	68/26	0.056
CEA (≥5ng/ml/<5ng/ml)	17/23	67/156	0.120	5/12	41/88	0.843	12/11	26/68	0.024
CA125 (≥35U/ml/<35U/ml)	10/30	39/184	0.261	4/13	26/103	0.746	6/17	13/81	0.153
Laparoscopic surgery (Yes/No)	9/31	56/167	0.724	5/12	33/96	0.735	4/19	23/71	0.470
Postoperative hospitalization stays (days), Median (IQR)	15(11-21)	13(10-16)	0.036	16(14-21)	12(10-15)	0.003	15(11-21)	13(11-17)	0.191
CR-POPF (Yes/NO)	14/26	34/189	0.003	5/12	12/109	0.152	9/14	14/80	0.009
Adjuvant chemotherapy (Yes/NO)	31/9	186/37	0.370	14/3	107/22	0.951	17/6	79/15	0.257

SD, standard deviation; FPR, fibrinogen/prealbumin ratio; BMI, body mass index; PTCD, percutaneous transhepaticcholangial drainage; AJCC, American Joint Committee on Cancer; TNM, tumor node metastasis; CA, carbohydrate antigen; CEA, carcinoembryonic antigen; IQR, interquartile range; CR-POPF, clinically relevant postoperative pancreatic fi.

### 3.4 Identification of independent risk factors for OS

**Figure 3:**
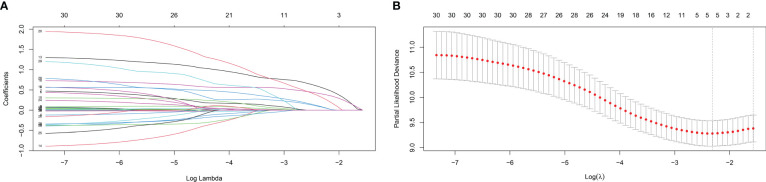
Variable selection using the least absolute shrinkage and selection operator (LASSO) regression model. **(A)** Coefficient profiles plot **(B)** Optimum parameter (lambda) selection.

In the training cohort, LASSO regression was used to select predictive variables from those shown in **Table 1** and five immune-nutritional markers. LASSO can avoid model overfitting. Tenfold cross-validation was performed to determine the optimal lambda value. According to the optimum value corresponding to the minimum value of lambda, five variables with nonzero coefficients were retained, including FPR, CONUT, CA199, CEA, TNM stage (**Figure 3**). The above variables were used for multivariable Cox regression analysis to identify the independent factors with a significant impact on OS. Finally, FPR≥0.29 (P=0.001, HR=2.592, 95%CI: 1.462-4.595), CONUT≥4 (P=0.015, HR=2.462, 95%CI: 1.187-5.105), CA199≥37U/ml (P=0.031, HR=1.648, 95%CI: 1.022-2.658), CEA≥5ng/ml (P=0.002, HR=1.958, 95%CI: 1.268-3.024) and TNM stage II (P=0.046, HR=1.531, 95%CI: 1.007-2.328) were identified to be independent risk factors for poor OS (**Table 3**).

**Table 3:** Univariate and multivariate analysis of risk factors for overall survival in training cohort.

Variables	Univariate analysis			Multivariate analysis		
	HR	95% CI	P Value	HR	95% CI	P Value
FPR ≥0.29	2.356	1.395-3.981	0.001	2.592	1.462-4.595	0.001
TNM stage						
II	1.494	1.010-2.211	0.045	1.531	1.007-2.328	0.046
III	2.359	0.726-7.669	0.154	2.294	0.692-7.603	0.175
CONUT ≥4	2.626	1.355-5.091	0.004	2.462	1.187-5.105	0.015
CA199 ≥37U/ml	1.526	0.994-2.344	0.054	1.648	1.022-2.658	0.031
CEA ≥5ng/ml	1.960	1.308-2.936	0.001	1.958	1.268-3.024	0.002

HR, hazard ratio; CI, confidence interval; FPR, fibrinogen/prealbumin ratio; TNM, tumor node metastasis; CONUT, controlling nutritional status; CA, carbohydrate antigen; CEA, carcinoembryonic antigen.

### 3.5 Construction and validation of nomogram

**Figure 4:**
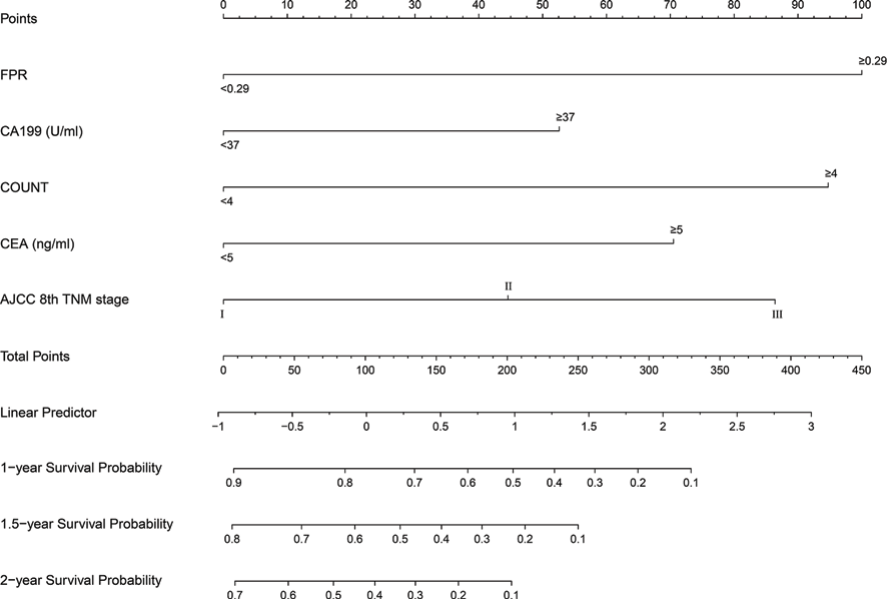
Survival nomogram for patients with resectable pancreatic cancer. To use the nomogram, an individual’s value is located on each variable axis, and a line is drawn upward to determine the number of points received for each variable. The sum of these numbers is located on the total points axis, and a line is drawn downward to the survival axes to determine the likelihood of survival at 1 year, 1.5 year and 2 years. FPR, fibrinogen-to-prealbumin ratio; CA, carbohydrate antigen; CEA, carcinoembryonic antigen; AJCC, American Joint Committee on Cancer; TNM, tumor node metastasis.

**Figure 5:**
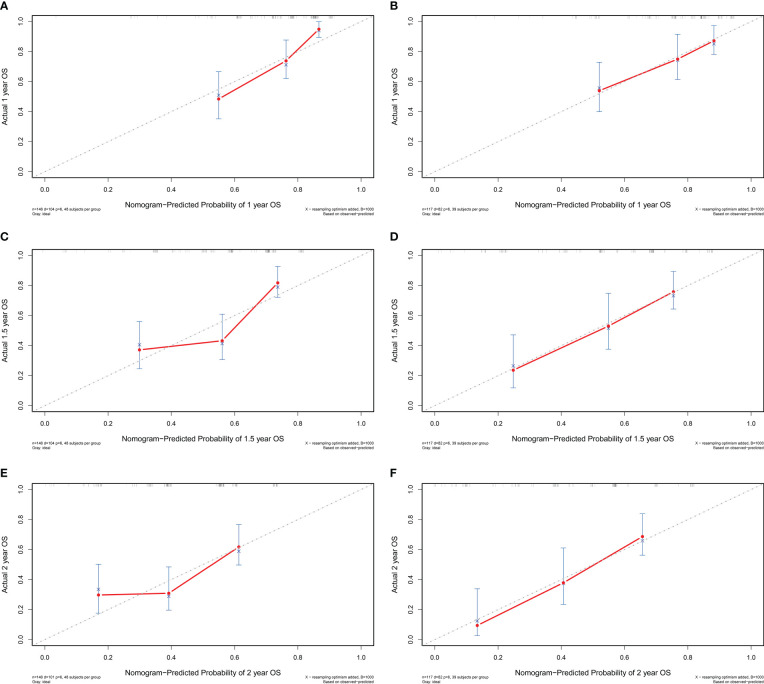
The calibration curve of the nomogram for 1-year, 1.5-year and 2-year survival of patients with resectable pancreatic cancer in the training cohort **(A–C)** and validation cohort **(D–F)**. OS, overall survival.

A prognostic nomogram was established by integrating above five significant independent risk factors for OS in the training cohort. Among these independent prognostic factors, FPR contributed the most to prognosis. Each variable was given a score on the points scale, and a total score was calculated from the nomogram. A higher total score indicated a shorter OS. The nomogram showed the predicted probabilities of 1-year, 1.5-year and 2-year survival (**Figure 4**). The C-index fitting model and calibration curves were used to estimate the predictive performance of the nomogram. The C-index of the training cohort and validation cohort were 0.703 (95% CI: 0.0.646-0.761) and 0.728 (95% CI: 0.671-0.784), which indicated the model had a good prediction ability. The calibration curves for survival probability in the training cohort and validation cohort were shown in **Figure 5**. The calibration curves were close to the diagonal line, which suggested that the prediction probability have good agreement with the actual probability.

### 3.6 Clinical benefit assessment of nomogram

**Figure 6:**
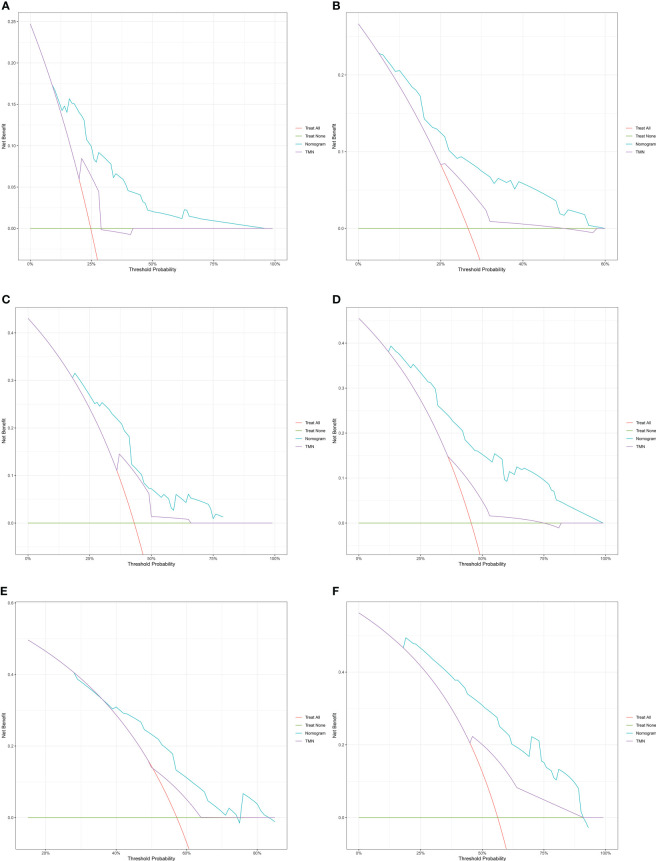
The decision curve analysis of the nomogram for 1-year, 1.5-year and 2-year survival of patients with resectable pancreatic cancer in the training cohort **(A–C)** and validation cohort **(D–F)**. TNM: tumor node metastasis.

**Figure 7:**
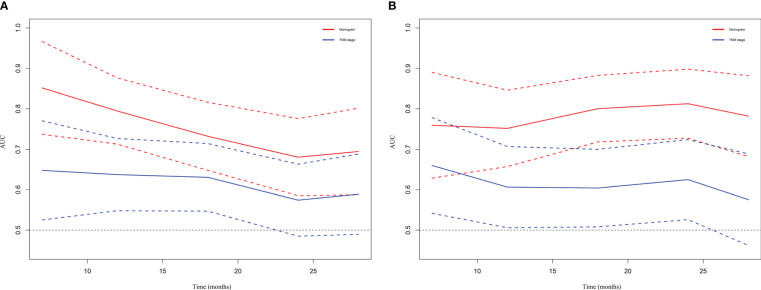
Time-dependent AUC plot for survival prediction in the training cohort **(A)** and validation cohort **(B)**. TNM, tumor node metastasis.

DCA and time-dependent AUC were performed to compare clinical benefit of the nomogram with that of conventional TNM stage. The 1-year, 1.5-year and 2-year DCA plots showed that the nomogram provided larger net benefit across the range of reasonable threshold probabilities than the TNM stage (**Figure 6**). The time-dependent AUC plots showed that the AUC of the nomogram fluctuated above 0.7 in both the training cohort and validation cohort. Compared with conventional TNM stage, the nomogram had better discriminative ability and comparative stability (**Figure 7**).

### 3.7 Risk stratification of OS

**Figure 8:**
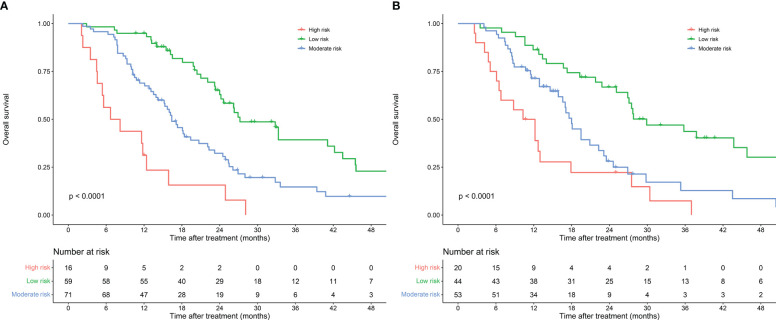
Risk stratification of nomogram model for patients with resectable pancreatic cancer in the training cohort **(A)** and validation cohort **(B)**.

A risk stratification was developed on the fundamental of scores calculated by the nomogram model to assess the clinical predictive ability of the nomogram model. Based on the scores of the patients in the training cohort, the optimal cut-off points of risk scores were calculated by X-tile software. Patients were classified into low-risk group (total points ≤ 81), moderate-risk group (81<total points ≤ 181) and high-risk group (total points>181) according to their risk scores. Kaplan-Meier survival curves showed significant different prognostic strata among patients in the training cohort (P<0.001) and the validation cohort (P<0.001) (**Figure 8**).

## 4 Discussion

For a long time, radical surgery has seemed to be the only hope for long-term survival in patients with pancreatic cancer. However, even treated with radical surgery, the overall survival time remains dismal (34). Accurate preoperative assessment of prognosis should be performed for each resectable pancreatic cancer patients to develop next treatment plan. If patients with poor prognosis could be identified preoperatively, individual preoperative therapy such as neoadjuvant treatment can be selected to improve prognosis. Nevertheless, there are limitations in predicting the prognosis of patients with pancreatic cancer after radical resection only by conventional TNM stage (7). Therefore, more biomarkers are needed to help guide therapy and improve the accuracy of prognostic determinations for individual patients. As a novel serological prognostic marker, multiple studies have demonstrated that FPR can evaluate the prognosis of malignant tumors such as colorectal cancer, hepatocellular carcinoma, esophageal cancer and gastric cancer (13–15, 35). A recent meta-analysis pointed out that high value of FPR is associated with poorer survival and increased risk of recurrence in cancer patients (36). However, there is still a lack of research on the role of FPR in the prognosis of resectable pancreatic cancer. Our study demonstrates for the first time that FPR is a feasible predictive marker for the prognostic assessment of resectable pancreatic cancer. Furthermore, we established a nomogram model incorporating FPR and other significant predictive factors to predict prognosis accurately.

This retrospective study investigated the prognostic value of FPR in patients with resectable pancreatic cancer and assessed the relationship with other clinicopathological factors. The analysis results showed that preoperative FPR increase is related to the poor prognosis of patients with resectable pancreatic cancer, patients with FPR≥0.29 had significantly shorter median survival time than FPR<0.29. Univariate and multivariate analysis revealed that FPR≥0.29 is an independent risk factor for a poor prognosis in patients with resectable pancreatic cancer. Compared with low FPR group, high FPR group had a significantly higher proportion of jaundice, pancreatic head tumors and CA199≥37U/ml. In addition, FPR was significantly associated with short-term prognosis in resectable pancreatic cancer, high FPR group tend to have longer hospital stays and a higher rate of CR-POPF. Pancreatic head cancer can lead to obstructive jaundice and has a heavier local inflammatory response. This can affect the patient’s nutritional status (37). Inflammatory responses and poor nutritional status increase the risk of postoperative adverse events such as CR-POPF (38). When jaundice and pancreatitis occur, CA199 levels may also be elevated (39).

Inflammatory response is closely related to the occurrence, development and resistance to radiotherapy and chemotherapy of pancreatic cancer (40, 41). In pancreatic cancer, cancer cells, stromal cells and inflammatory cells interact and participate in the composition of the inflammatory microenvironment (41). Fibrinogen is an inflammation-related acute phase reactant that exists in the tumor microenvironment of pancreatic cancer and is closely related to the biological behavior of the tumor (25, 42). Fibrinogen synthesis is markedly increased in pancreatic cancer patients (43). Fibrinogen can promote the production of interleukin 6, which is involved in the inflammatory response and causes tumor progression (44). Under the action of thrombin, fibrinogen in the tumor stroma is converted into insoluble fibrin, which provides adhesion sites for endothelial cells and tumor cells (45). In addition, fibrinogen can interact with platelets, hinder the killing of tumor cells by natural killer cells, and cause tumor immune escape (46). A recent study reported that preoperative elevated levels of plasma fibrinogen can predict distant metastasis of pancreatic cancer (47). Another study also pointed out that hyperfibrinogen correlates with systemic inflammatory response and can predict poor prognosis in advanced pancreatic cancer (48).

Nutritional status is an important prognostic factor for cancer patients (49, 50). Especially in pancreatic cancer patients, malnutrition is prevalent and associated with tumor cachexia (51, 52). Prealbumin is an important serological nutritional marker. Compared with albumin, it has a shorter half-life and is more sensitive to the fluctuation of the body’s nutritional status (20). Additionally, prealbumin is also an acute phase negative protein, and its synthesis will be reduced under inflammation (16, 17). Previous studies have shown that low prealbumin levels was closely associated with poor prognosis in cancer patients (53, 54).

Although both fibrinogen and prealbumin have been confirmed to be associated with the prognosis of cancer patients, remarkably, not all patients have abnormalities in both indicators at the same time. Therefore, combining the two indicators into an FPR can more significantly magnify the effect of a single indicator. Higher FPR suggests worse nutritional status and more severe inflammation, allowing for a more comprehensive assessment of prognosis.

Besides FPR, several other immune-nutritional markers were confirmed to be associated with poor prognosis of pancreatic cancer such as FAR (10), NLR (31), PNI (11), CONUT (12). Our study confirmed that FPR had comparable prognostic accuracy with above-mentioned indicators. Because of the limited predictive power of individual indicators, we established a predictive nomogram that integrated FPR with four other independent prognostic factors (CONUT, CA199, CEA, TNM stage). After validation, nomogram showed favorable ability of survival prediction, with a C-index of 0.703 in the training cohort and 0.728 in the validation cohort, and its prediction accuracy and clinical validity are better than that of the AJCC 8^th^ TNM staging system. Moreover, we constructed a useful risk-stratification model based on a nomogram, which can be useful for differentiating patients of different risk groups to better provide individualized treatment decision and surveillance. All indicators involved were routine preoperative examinations, and their measurements are convenient and inexpensive. Our model provides a useful tool which could help clinicians recognize high-risk patients and devise an optimal treatment plan. When the risk estimates of poor prognosis after surgery are at high-risk levels, the treatment plans of these patients should be adjusted in time. If necessary, the operation can be appropriately postponed and nutritional support or neoadjuvant treatment can be given before surgery to improve the prognosis of the patients.

There are also several limitations to this research. First, this is a single-center retrospective study with a relatively small sample size. The study may have been prone to selection bias. Second, some potential prognostic markers of pancreatic cancer like c-reactive protein were not considered in the model because they were not routine preoperative tests in our institution. Therefore, larger multicenter studies and more indicators are needed to further validate the prognostic value of prognostic model.

In conclusion, our study demonstrates that preoperative FPR is an independent prognostic factors affecting the prognosis of patients with resectable pancreatic cancer and established a new nomogram. Compared to conventional TNM stage, our nomogram showed more adequate discriminative ability and higher predictive accuracy. which can provide a useful tool for clinicians in making individualized treatment strategies and survival predictions.

## Data availability statement

The original contributions presented in the study are included in the article/**Supplementary Material**. Further inquiries can be directed to the corresponding authors.

## Ethics statement

The studies involving human participants were reviewed and approved by Ethics Committee of Qilu Hospital of Shandong University. The patients/participants provided their written informed consent to participate in this study.

## Author contributions

Conceptualization: CL and ZF. Methodology: CL. Software: XM. Validation: WG and FLia. Formal analysis: HW and JW. Investigation: JX and HL. Resources: LW. Data curation: CL and ZF. Writing—original draft preparation: CL. Writing—review and editing: LW and FLi. Supervision: FLi. Project administration: LW. Funding acquisition: DW and FLi. All authors have read and agreed to the published version of the manuscript. All authors contributed to the article and approved the submitted version.

## Supplementary material

Supplementary Figure 1 Kaplan–Meier curves of overall survival in patients with resectable pancreatic cancer stratified by fibrinogen-to-prealbumin ratio (A), fibrinogen-to-albumin ratio (B), neutrophil-to-lymphocyte ratio (C), prognostic nutritional index (D), controlling nutritional status (E);

Supplementary Figure 2 Time-dependent AUC plot for survival prediction of immune-nutritional markers.
