# Supplementary material for: Fibrinogen-to-prealbumin ratio: A new prognostic marker of resectable pancreatic cancer
Source: Front Oncol. 2023 Mar 27;13:1149942. doi: 10.3389/fonc.2023.1149942 (PMC10083287; doi:10.3389/fonc.2023.1149942)

| **Supplementary Table 1 CONUT** **score** | | | | |
| --- | --- | --- | --- | --- |
| Variables | Normal | Mild | Moderate | Severe |
| Serum albumin (g/dL) | ≥ 3.50 | 3.00–3.49 | 2.50–2.99 | < 2.50 |
| Score | 0 | 2 | 4 | 6 |
| Lymphocyte count (/mm^3^) | ≥ 1600 | 1200–1599 | 800–1199 | < 800 |
| Score | 0 | 1 | 2 | 3 |
| Total cholesterol (mg/dl) | ≥ 180 | 140–179 | 100–139 | < 100 |
| Score | 0 | 1 | 2 | 3 |
| CONUT score = serum albumin score + lymphocyte count score + total cholesterol score | | | | |
| Total Score | 0-1 | 2-4 | 5-8 | 9-12 |

CONUT: controlling nutritional status

**Supplementary Figure 1** Kaplan–Meier curves of overall survival in patients with resectable pancreatic cancer stratified by fibrinogen-to-prealbumin ratio (A), fibrinogen-to-albumin ratio (B), neutrophil-to-lymphocyte ratio (C), prognostic nutritional index (D), controlling nutritional status (E).


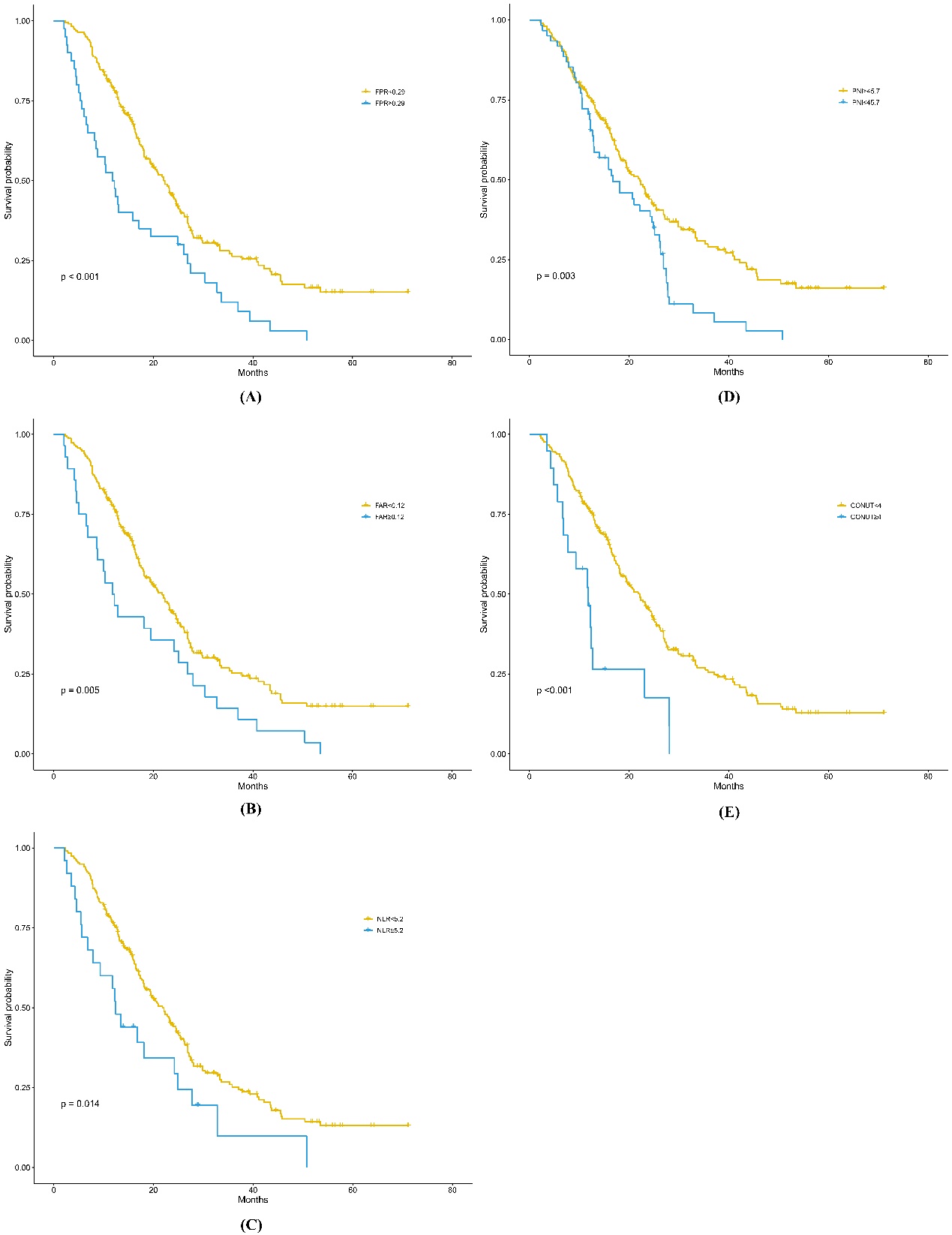


**Supplementary Figure 2** Time-dependent AUC plot for survival prediction of immune-nutritional markers. FPR: fibrinogen/prealbumin ratio; CONUT: controlling nutritional status; FAR: fibrinogen-to-albumin ratio; NLR: neutrophil-to-lymphocyte ratio; PNI: prognostic nutritional index.


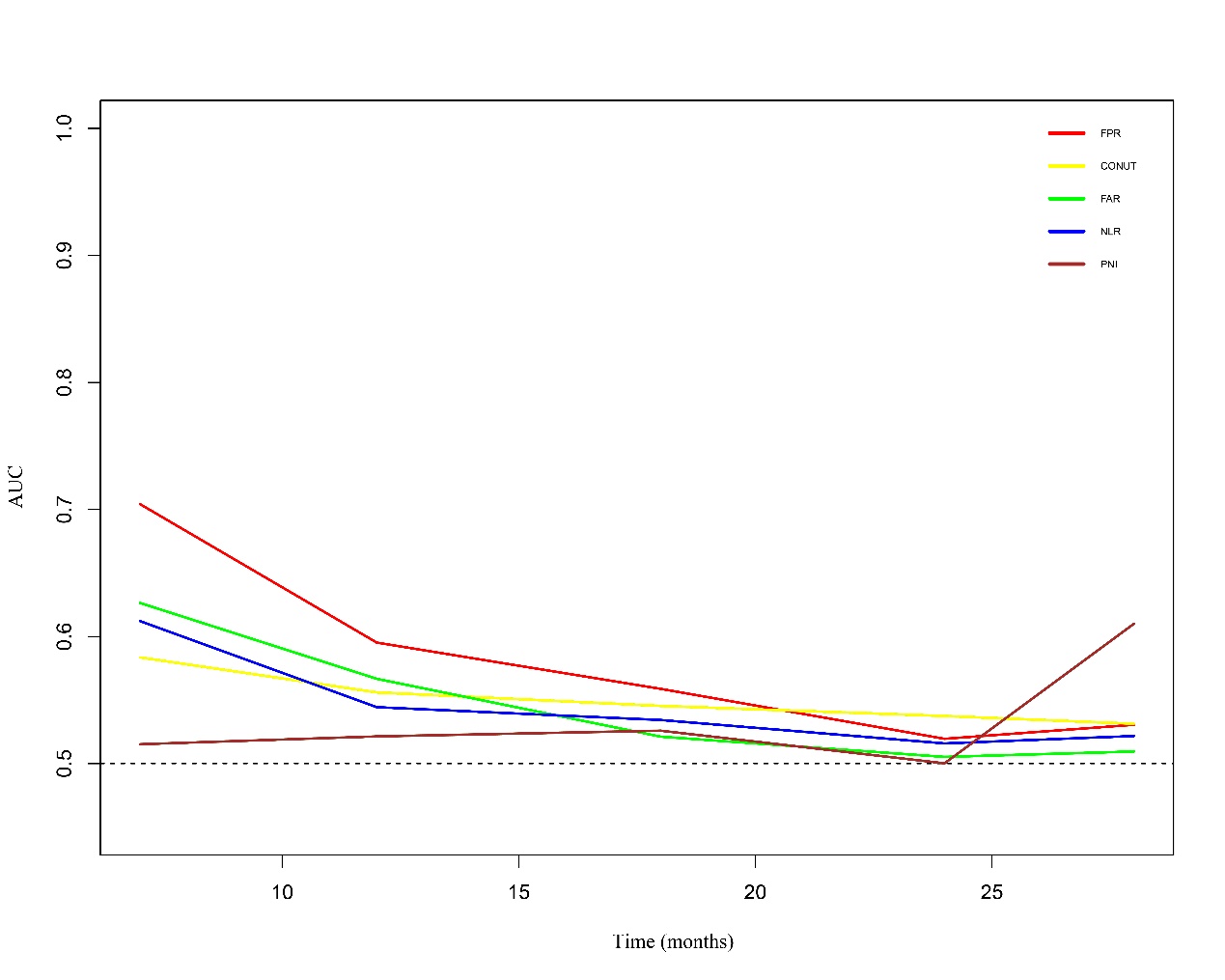

Supplement: Supplementary Figure 1 — Kaplan–Meier curves of overall survival in patients with resectable pancreatic cancer stratified by fibrinogen-to-prealbumin ratio (A), fibrinogen-to-albumin ratio (B), neutrophil-to-lymphocyte ratio (C), prognostic nutritional index (D), controlling nutritional status (E); [file DataSheet_1.docx]
